# Prostration of the Circulation

**Published:** 1904-05

**Authors:** Louis Faugeres Bishop

**Affiliations:** New York


### Prostration of the Circulation.

BY LOUIS FAUGERES BISHOP, A. M., M. D., New York.

THIS term is used as synonymous in reference to the circulatory system with neurasthenia in relation to the nervous system. Neurasthenia is undoubtedly founded upon a malnutrition or an exhausted vitality of nervous tissue. Prostration of the circulation is founded upon the corresponding elements, but as the heart and bloodvessels are much more open to direct examination than the nerves, there are signs as well as symptoms. As in neurasthenia both the brain and the peripheral nerves participate in the disease, so in prostration of the circulation the heart and bloodvessels are involved. Neurasthenia and prostration of the circulation are two conditions that have many correlations. In neurasthenia there is always a certain amount of vasomotor disturbance, though there are many cases in which the circulation is entirely normal.

In prostration of the circulation there is often much depression, but disturbances of sensation have a real foundation in alterations of the blood supply, and there are many cases in which there is not the slightest touch of neurasthenia.

I have suggested the name, Prostration of the circulation, to cover those cases in which the terms myocarditis and arterial degeneration are too harsh, just as we would not like to speak of neurasthenia as insanity. We have for a long time needed a word to cover those cases which present disturbance of function, and which are perfectly capable of complete recovery, though as often as not they go on to the manifestation of organic disease.

The following case which I saw recently will illustrate the point I wish to make. The case was that of a woman who had worked very hard all her life, had become run down and began to find that it was difficult for her to carry on her occupation. She presented the appearance of health; there were no evidences of kidney disease and no history of dissipation. She complained of some shortness of breath on exertion, particularly when there was any sudden demand upon her muscular system. She was not in the least neurasthenic, for these conditions were perfectly real in their existence. The arterial pulse was soft and compressible; her heart sounds were feeble and there was a soft systolic murmur at the apex.

It seemed to me a simple case of circulatory prostration and one that was capable of complete recovery by a suitable course of treatment. It is not easy to describe the differentiation of such a case from a case of real myocardial degeneration, or a case of circulatory disease in which the kidneys may eventually be the principal sufferers. However, there is this about it—namely, that in myocarditis, irregularity of force and rhythm are a very constant symptom, and in commencing arterial degeneration careful search is made to discover some signs of irregularity of cerebral circulation. Cases of prostration of the circulation show marked hepatic symptoms, principally due to the stagnation of the circulation in the liver. In other words, in simple prostration of the circulation the blood current is feeble but regular. In myocarditis and arterial degeneration it is irregular.

54 West Fifty-fifth Street.

